# Secondary Leukemia in a non-Hodgkin's Lymphoma Patient Presenting as Myeloid Sarcoma of the Breast

**DOI:** 10.1155/2011/914613

**Published:** 2011-07-12

**Authors:** Vincenzo Pitini, Carmela Arrigo, Maria Grazia Sauta, Giuseppe Altavilla

**Affiliations:** Department of Medical Oncology, University of Messina, Via Consolare Valeria, 98125 Messina, Italy

## Abstract

As defined by the World Health Organization classification of tumors of hematopoietic and lymphoid tissue, myeloid sarcoma (MS) is a tumor mass of myeloblasts or immature myeloid cells that can arise before, concurrent with, or following acute myeloid leukaemia. We describe a case of secondary leukemia presenting itself as MS of the breast in a patient previously treated for a non-Hodgkin's Lymphoma.

## 1. Introduction

Myeloid sarcoma (MS) is a localized extramedullary tumor composed of immature myeloid cells. It is usually seen in association with acute leukemia, myeloproliferative neoplasm, and myelodysplastic syndromes. It can also occur *de novo* in a patient with a normal bone marrow, and no prior history of acute myeloid leukemia (AML) [1]. These tumors have a predilection for the skin, lymph nodes, small intestine, spine, and the mean time between the diagnosis and development of AML in the largest number of patients who had no evidence of leukemia at presentation was 10.5 months [2]. In addition MS may be the initial manifestation of relapse of AML following chemotherapy or hematopoietic stem-cell transplantation [3–5]. Many cases of MS are initially misdiagnosed as malignant lymphomas. Here we report a case of myeloid sarcoma of the breast in a patient previously treated for a non-Hodgkin's Lymphoma.

## 2. Case Report

**Figure 1:**
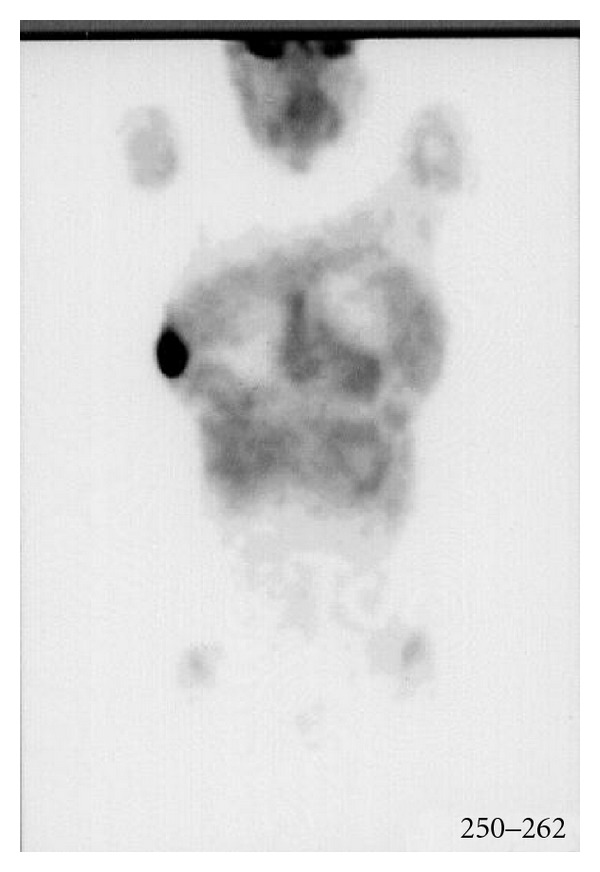
FDG-PET: positive uptake of radiotracer in the right breast.

**Figure 2:**
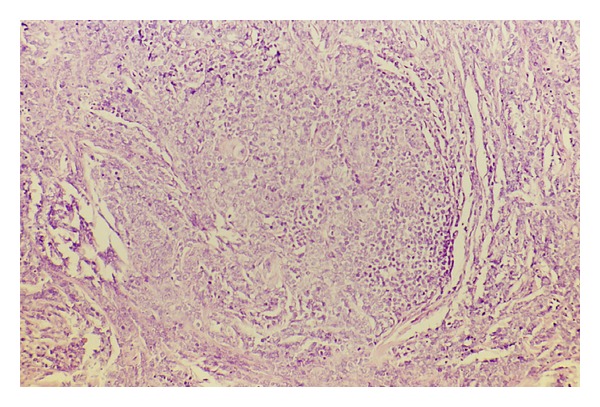
Neoplastic population basically formed by blasts.

**Figure 3:**
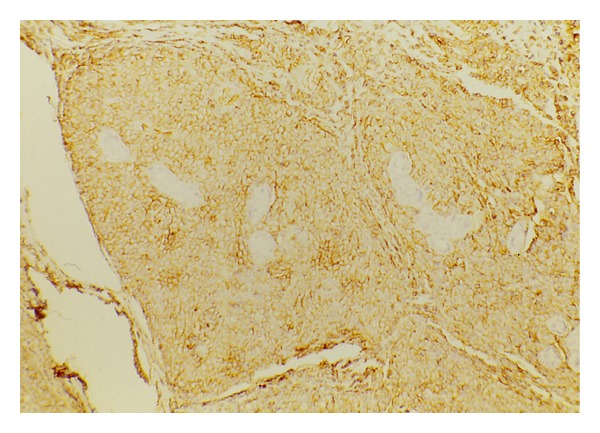
CD34: intense positive immunostaining.

A 35-year-old female was referred to our department because of a breast nodule in the upper right region. One year earlier, she had been diagnosed with stage IV B mediastinal large B-cell lymphoma. Subsequently the patient was treated with high-dose sequential (HDS) chemotherapy, followed by an autologous stem-cell transplantation with complete remission. The HDS is a comprehensive program including an initial debulking with two APO, two DHAP, and an high chemotherapy phase with PBPC harvesting followed by an autologous stem cell transplantation [6]. Initial investigations included a computed tomography scans of the abdomen, thorax, and pelvis which failed to detect any further abnormality, a whole body positron emission tomography using ^18^F-fluorodeoxyglucose (FDG-PET) showed a positive uptake of radiotracer in the right breast suggesting a recurrent disease (Figure 1). On examination a firm, slightly mobile mass with skin thickening was palpated in the upper outer right quadrant, no enlarged lymph nodes were found in the right axillary region, no other significant physical examination findings were noted. A mammography revealed a solitary ill-defined mass in the upper outer quadrant of the right breast. The lesion was surgically removed. At light microscopy, a tumoral growth which diffusely infiltrated the mammary gland consisting mainly of blasts with variably shaped nuclei, multiple evident nucleoli, a rather dense chromatin and a moderate amount of cytoplasm was demonstrated (Figure 2). Surprisingly, immunohistochemistry showed that the neoplastic cells were strongly stained for CD34 (Figure 3) and myeloperoxidase, being negative for the remaining cell markers employed CD79a, CD20, CD10, CD3, and Glycophorin A. Based on morphologic and phenotypic findings a diagnosis of myeloid sarcoma was made. Following the histological diagnosis of acute myeloproliferative disorder, the patient underwent extensive laboratory investigations which showed normal red blood cell, white blood cell, and platelet counts with no blasts in the peripheral blood. Bone marrow aspirates and trephine biopsies displayed neither significant abnormalities nor evidence of leukemic infiltration and a normal karyotype. Due to a low yield of assessable metaphase cells from the right breast lesion a comprehensive analysis of the most relevant AML-associated chromosome aberrations was performed in the smears from the lesion and on formalin fixed paraffin embedded MS tissue sections by fluorescence in situ hybridization. Molecular cytogenetic analysis using DNA probes revealed a complex karyotype that included trisomy 8 (77%), monosomy 7 (67%), and a del 11q23 (59%).

On the basis of these findings we decided to treat our patient with radiotherapy (total dose 3.600 cGy), and two courses of high-dose ARA-C even in the persistent absence of detectable medullary tumor after radiotherapy, since she did not have a sibling match for an allogenic transplant. Nevertheless, the disease had a relapse after 5 months and pursued a fulminant course causing the patient's death.

## 3. Discussion

Therapy-related myelodysplasia (tMDS) and secondary acute myelogenous leukemia (sAML) are well-recognised late complications of high-dose therapy for lymphoid malignancies (incidence between 5% and 15%). tMDS and sAML are clinically and cytogenetically distinct from *de novo* cases and the observed cytogenetic changes are often characteristic of chemotherapy-induced chromosomal damage [7]. Alkylating agents are associated with complete or partial deletions of chromosome 5 and/or 7. Topoisomerase II are typically associated with balanced translocations involving chromosome bands 11q23 and 21q22. Our case had characteristics of both the major groups of therapy-related leukemia even if a very short latency period after chemotherapy (12 months) was observed. 

Our case is instructive at a number of levels: firstly, for the very uncommon clinical presentation; secondly, it highlights the need to fully integrate morphologic, immunophenotypic, and genetic data to reach specific diagnoses, even if they are rather unusual.
